# Yeasts for Sustainable Agriculture: Integrating Plant Growth Promotion and Phytopathogen Biocontrol

**DOI:** 10.1155/ijm/2832069

**Published:** 2026-10-22

**Authors:** Mariajose Carvajal, Javiera Ferreira, Carlos Silva-Medina, Liliana Godoy

**Affiliations:** ^1^ Departamento de Fruticultura y Enología Facultad de Agronomía y Sistemas Naturales Pontificia Universidad Católica de Chile Santiago Chile uc.cl

**Keywords:** biocontrol, biostimulant, phytopathogen, plant growth–promoting yeasts, yeast

## Abstract

Although microbial bioproducts are part of sustainable agriculture, research and commercialization have focused mainly on rhizobacteria and mycorrhizal fungi, leaving yeasts comparatively underexplored relative to other microbial groups. Where documented, several yeast species have been associated with enhanced plant performance through phytohormone synthesis, nutrient solubilization, and ACC‐deaminase activity, as well as with suppression of fungal pathogens via competition for resources, lytic enzymes, killer toxins, volatile organic compounds (VOCs), mycoparasitism, and elicitation of plant defense responses. However, the strength of evidence supporting these mechanisms varies considerably. Many reported effects have been characterized primarily in vitro or in postharvest systems; field‐level efficacy data across diverse agroecological zones remain limited, and the molecular modes of action underlying yeast–plant interactions are only partially understood. Claims of improved salinity and drought tolerance, hormone‐mediated growth promotion, and systemic resistance induction remain largely associative and require direct confirmation under field conditions. This review critically synthesizes recent advances in yeast biology relevant to sustainable agriculture, evaluates the strength of available evidence across experimental scales, and inventories commercial live‐yeast products. It identifies five priority research areas needed to bridge the gap between laboratory promise and reliable agricultural application of yeasts as dual‐purpose biostimulants and biocontrol agents.

## 1. Introduction

Microbial bioproducts have gained increasing attention as potential tools for agricultural applications that (i) improve nutrient‐uptake efficiency, (ii) mitigate abiotic stresses, and (iii) suppress pests and pathogens. Commercial formulations rely mostly on bacteria such as *Bacillus* spp. or filamentous fungi such as *Trichoderma* spp., which are well‐established plant growth–promoting agents [1], predominantly composed of *Bacillus*, *Pseudomonas*, *Streptomyces*, and *Rhizobia* among bacteria [2–4], or *Trichoderma* among fungi [5, 6]. Despite being comparatively less studied, yeasts hold considerable significance in the biostimulant industry, where only a few products are currently derived from them.

Yeasts can consume simple organic carbon compounds, vitamins, and organic acids released from root exudation [7], and some species possess a facultative anaerobic metabolism that allows switching between fermentation and respiration depending on ambient conditions. Medina et al. [8] introduced the term plant growth–promoting yeasts (PGPYs) in 2004 while studying *Yarrowia lipolytica*. Since then, *Candida*, *Rhodotorula*, *Cryptococcus*, and *Saccharomyces* have emerged as the genera most frequently reported as PGPYs [9]. Yeasts have been reported as plant growth–promoting microorganisms.

Several mechanisms contribute to this trait, including the solubilization of nutrients for the synthesis of plant hormones, the emission of volatile organic acids, and 1‐aminocyclopropane‐1‐carboxylate deaminase (ACCD) activity [10–13]. In addition, yeasts can act as biocontrol agents by activating induced systemic resistance (ISR), generating enzymes, and producing volatile organic compounds (VOCs) [14], among others [15]. Despite these characteristics, there are few studies on living yeasts as biostimulants. Overall, the reported results indicate that yeasts could be considered as potential components in the development of new biostimulants [15, 16].

Yeasts have been proposed as complementary tools to conventional fertilizers and fungicides, with reported benefits for plant growth promotion and postharvest disease management. Studies have documented improvements in crop yield and quality following yeast inoculation under controlled conditions [17, 18], and yeast‐based biofungicides have shown efficacy against postharvest fungal diseases in fruits and vegetables, particularly in controlled and semicommercial settings [19] (Figure 1). However, the consistency of these effects under open field conditions and across diverse agroecological contexts remains to be established.

**Figure 1:**
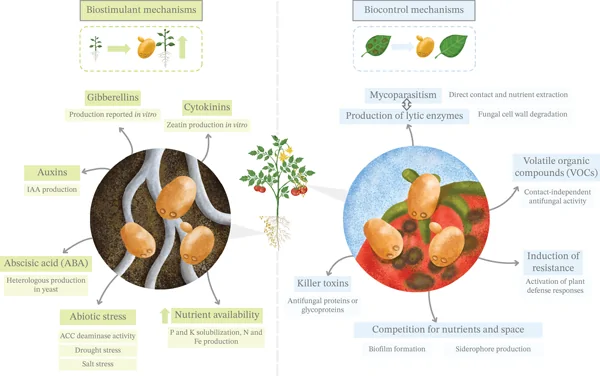
Biostimulant and biocontrol mechanisms mediated by yeasts. Conceptual overview summarizing major mechanisms attributed to yeasts, based primarily on laboratory, greenhouse, and postharvest studies. The relevance and contribution of each mechanism may vary depending on yeast strain, host plant, and environmental context. On one side, the figure illustrates how yeasts could promote plant growth and enhance abiotic stress tolerance, whereas on the other, it depicts the biocontrol mechanisms yeasts employ against various plant pathogens. Abbreviations: IAA, indole‐3‐acetic acid; ABA, abscisic acid; ACC, 1‐aminocyclopropane‐1‐carboxylate; P, phosphorus; K, potassium; N, nitrogen; Fe, iron; VOCs, volatile organic compounds.

This review summarizes the different biostimulant and biocontrol mechanisms and modes of action of yeasts described in previous studies (Figure 1). Additionally, it explores the current situation regarding yeast application in the agricultural industry and discusses available alternatives. Ultimately, yeasts should be included among biostimulant microorganisms that contribute to plant growth–promoting traits and biocontrol.

Unlike previous reviews that focus predominantly on cataloging yeast species or applications, this review emphasizes a critical evaluation of mechanistic evidence, clearly distinguishing between laboratory‐based findings and validated plant‐level responses, and highlighting limitations, inconsistencies, and research gaps that currently constrain large‐scale agricultural implementation.

Ultimately, the objective of this narrative review is to provide a broad, updated, and critical synthesis of the role of yeasts in sustainable agriculture, highlighting their mechanisms of action both as biostimulants and as biocontrol agents.

## 2. Methodology

This review was conducted through a literature search in indexed journals and databases including Scopus, Web of Science, and Google Scholar. Only peer‐reviewed articles published between 2010 and 2026 were considered. Additional papers and books outside this time frame were included when they provided definitions or represented seminal findings in the field. Only articles published in English were reviewed, and nonpeer‐reviewed sources such as theses and preprints were excluded. Selection criteria encompassed reviews on biostimulant microorganisms, yeast strains with biostimulant or biocontrol activity, scientific articles reporting in vitro and in vivo experiments, and studies describing yeast‐based products that had been scientifically tested. Keywords employed in the search included biostimulant yeasts, biocontrol yeasts, PGPYs, agrochemical products based on yeast, and yeast products in agricultural production, among others.

The information was initially classified according to reports of biostimulant and biocontrol activities, including the mechanisms described and the corresponding screenings and experiments that validated their effects on plant growth promotion or phytopathogen control. Subsequently, reports on molecular pathways underlying yeast mechanisms of action were analyzed, and a summary table was compiled. Finally, a critical perspective was provided on the implications and applications of yeasts in agroindustry, including reports of existing yeast‐based products and a discussion addressing laboratory versus greenhouse versus field evidence, strain‐specific versus generalizable effects, and confirmed mechanisms versus proposed hypotheses.

Limitations of this review include the potential for selection bias, as it is not a systematic synthesis. Nevertheless, it is aimed at delivering a comprehensive and critical overview of the topic.

## 3. Biostimulant Mechanisms of Yeasts

The mechanisms described in this section are supported by evidence of markedly different strength and origin. Nutrient solubilization and ACCD activity are the most consistently documented across in vitro, greenhouse, and in a smaller number of cases, field studies. Phytohormone synthesis (auxins, cytokinins [CKs], gibberellins [GA], and abscisic acid [ABA]) has been demonstrated mainly through in vitro screening assays and heterologous expression systems, with in planta hormone quantification available for only a small subset of reported cases. Throughout this section, we indicate where a finding has been confirmed at the plant or field level versus where it remains an in vitro observation or an extrapolation from nonyeast microbial systems, following the evidence hierarchy detailed in Section 7. Figure 1 summarizes the mechanisms by which yeasts may promote plant growth and enhance tolerance to abiotic stress.

### 3.1. Nutrient Availability

#### 3.1.1. Phosphorus

Phosphorus is an essential nutrient for plants, playing a crucial role in energy transport, DNA composition, and carbohydrate synthesis. Generally, only 5% of the total amount of phosphorus is available for plant uptake.

Among microorganisms, yeasts have been reported to exhibit phosphatase activity. These microorganisms can solubilize phosphorus by breaking down phosphorus‐containing compounds, releasing phosphate in a plant‐accessible form [12]. Two primary mechanisms have been described for how yeasts mobilize immobilized phosphorus. The first involves the exudation of protons or low‐molecular‐weight organic acids, which facilitate the dissociation of precipitated inorganic phosphorus by acidifying the environment or chelating associated metal cations, such as calcium, magnesium, or iron. This process also promotes the release of orthophosphate adsorbed to soil particles through ligand‐exchange reactions. The second mechanism involves the secretion of phosphatase enzymes, which catalyze the hydrolysis of organic phosphorus compounds, thereby making inorganic phosphate (Pi) available for plant uptake [20]. Ultimately, this ability to mobilize phosphorus is considered an adaptive response to surviving in environments with limited soluble inorganic P availability [21, 22].

Reported yeast species with this nutrient solubilization ability are *Candida* spp., *Rhodosporidium paludigenum*, *Cryptococcus laurentii*, *Torulaspora* spp., *Candida tropicalis*, *Wickerhamomyces anomalus*, *Mrakia psychrophila*, among others. Yeasts can solubilize insoluble Pi under varying concentrations of soluble phosphorus. Kuo et al. [23] demonstrated that the presence of soluble phosphorus did not inhibit the solubilization process in some species of yeasts.

To explore yeast phosphorus‐solubilizing activity in a soil matrix, Yan et al. [24] evaluated two distinct Chinese soils, a natural red soil and a continuously fertilized paddy soil. Following the incubation of soil samples with a *Candida mycoderma* inoculum, the authors observed that the yeast reduced soluble phosphorus levels in the red soil, whereas increased them in the paddy soil. These findings suggest that such contrasting behavior is dictated by the specific pH and organic matter content of each soil, which jointly determine the yeast′s capacity to grow and function. Because the soil was sterilized and no living plants were present, however, these results reflect the yeast′s intrinsic solubilizing activity under controlled conditions rather than its behavior in an intact, plant‐associated soil ecosystem, and the general dynamics of yeasts in complex soil ecosystems remain poorly understood.

#### 3.1.2. Nitrogen (N_2_)

Nitrogen is an essential nutrient required in large quantities by plants. It is most commonly supplied through synthetic fertilizers, such as urea, which is produced via chemical nitrogen fixation. In contrast to other microorganisms known to fix atmospheric nitrogen through the activity of nitrogenase by transforming it into ammonia, the capacity of yeasts to fix N_2_ remains poorly understood [25].

Conversely, NH_3_ production by yeasts is more common and has been reported across numerous species. Yeasts likely excrete NH_3_ as a signaling molecule for intercellular communication between colonies [26]. The release of NH_3_ into the soil is essential for plant nutrition: It is rapidly protonated to ammonium (NH_4_^+^) or enters the nitrification pathway, where it is oxidized to nitrite by *Nitrosomonas* and then to nitrate (NO_3_^−^) by *Nitrobacter*. Ultimately, both NH_4_^+^ and NO_3_^−^ serve as readily available nitrogen sources for plant assimilation [27].

Several studies have isolated yeasts from plant rhizospheres capable of ammonia production even in nitrogen‐free media. The most prolific ammonia‐producing genera include *Candida*, *Cryptococcus*, *Debaryomyces*, *Papiliotrema*, *Pichia*, and *Rhodotorula* [10, 28, 29]. Moreover, literature suggests that nitrogen fixation by these yeasts occurs exclusively under specific conditions: the presence of a carbon source (e.g., sugars) alongside essential nutrients, coupled with the complete absence of external nitrogen. Whenever assimilable nitrogen becomes available in the environment, the fixation pathway is actively suppressed [30].

In this context, interactions that enhance plant growth under nitrogen deficiency conditions have been linked to the expression of nitrate transporter genes. For instance, a recent study demonstrated that three VOCs (isopropyl alcohol, 2‐pentylfuran, and 1,3‐pentadiene) produced by the yeast *Solicoccozyma terrea* modulate the expression of these genes, thereby stimulating lateral root formation in tomato plants [31].

Several microorganisms, such as *Trichoderma* spp., are well known for their ability to enhance plant growth through VOC emission, affecting both shoot and root development [32, 33]. In contrast, evidence for a similar effect in yeasts is limited to a few studies that suggest a biostimulant role of yeast‐derived VOCs, with the specific compounds and mechanisms remaining poorly defined [34–36]. Furthermore, although studies indicate that VOCs alone can stimulate plant growth, direct inoculation of microorganisms into the same medium may conversely lead to competition for nutrients and space, ultimately reducing root development. Therefore, it is advisable to evaluate both approaches experimentally, as the effectiveness of VOC‐mediated stimulation versus direct inoculation may vary depending on the microbial strain, plant species, and specific growth conditions [35].

#### 3.1.3. Potassium (K)

An essential macronutrient responsible for osmotic regulation in plants, stomatal opening, and balancing the negative charges of organic molecules. Although this element is found mainly in insoluble form in the soil, some microorganisms can solubilize it.

Several yeast species have been reported to exhibit potassium solubilizing capabilities. For instance, *Naganishia* sp. exhibited a potassium solubilization index of 2.5 cm when grown in a selective medium, which correlated with an increased potassium concentration [37]. Similarly, in experiments utilizing rock powder as a substrate, *Torulaspora globosa* exhibited notable potassium solubilization activity [38]. Additionally, in an in vitro experiment, *Pichia anomala* and *Rhodotorula glutinis* were shown to solubilize potassium from muscovite. When these yeasts were applied in a pot experiment with maize, the yeast treatments resulted in increased dry weight compared with the control [39].

#### 3.1.4. Iron

An essential micronutrient required for the formation of heme groups in cytochromes and nitrate reductase, thereby playing a critical role in electron transfer processes [40]. Despite its abundance, iron is often not readily available for plants due to its inherently low soil solubility.

To overcome this limitation, three main iron acquisition strategies have evolved. The first, typical of dicotyledonous and nongrass monocotyledonous species, involves rhizosphere acidification via H^+^‐ATPase activity, reduction of Fe^3+^ to Fe^2+^ by ferric chelate reductases, and subsequent cellular uptake by the iron‐regulated transporter 1 (IRT1). The second strategy, predominantly found in grasses, relies on the secretion of phytosiderophores (PS). These compounds chelate soil Fe^3+^ to form stable Fe^3+^‐PS complexes, which are subsequently internalized by specific transporters such as yellow stripe 1/yellow stripe‐like (YS1/YSL), a crucial mechanism for iron acquisition in alkaline soils where Fe^3+^ solubility is severely restricted. Furthermore, a third strategy involves synergistic plant–microorganism interactions: within the rhizosphere, microorganisms, including yeasts, secrete siderophores that bind Fe^3+^. By mimicking plant‐derived chelators, these microbial siderophores significantly enhance iron availability to the host plant under specific conditions [41].

Furthermore, certain yeasts from the genera *Metschnikowia* and *Rhodotorula* have been reported to secrete siderophores into the environment to form stable iron chelates. This secretion serves as a competitive ecological strategy, effectively sequestering iron from other microorganisms that are unable to take up these bulky iron–siderophore complexes [11]. Ultimately, this microbial activity indirectly biostimulates plant growth, as roots can absorb these chelated iron forms via pathways analogous to their intrinsic iron acquisition strategies.

### 3.2. Synthesis of Plant Hormones

#### 3.2.1. Auxins

Although yeasts are known to produce various plant hormones, auxin biosynthesis remains the most extensively studied. Auxins are crucial plant growth regulators involved in morphogenesis, root architecture development, and cellular elongation and division [42]. In plants, indole‐3‐acetic acid (IAA) is synthesized via both tryptophan (Trp)‐dependent and Trp‐independent pathways. Trp‐dependent routes involve intermediates such as indole‐3‐pyruvic acid (IPA), indole‐3‐acetamide (IAM), indole‐3‐acetonitrile (IAN), and tryptamine (TAM), with the IPA and IAM pathways being the most predominant under physiological conditions. Conversely, Trp‐independent pathways generate IAA directly from indole or carbon metabolism‐derived compounds [43, 44].

Among microorganisms, bacterial IAA biosynthesis is the most well characterized, encompassing the IAM, IPA, TAM, IAN, and tryptophan side‐chain oxidase (TSO) pathways. Furthermore, Trp‐independent IAA production has been documented, with tryptophol and indole‐3‐lactate proposed as potential precursors, though the exact mechanisms remain unconfirmed [45, 46]. In contrast, homologous metabolic pathways in yeasts remain comparatively underexplored. A study on *Rhodosporidiobolus fluvialis* primarily identified the IPA, IAM, and TAM pathways. Interestingly, although Trp‐independent IAA production by yeasts has been observed, the specific metabolic routes orchestrating this process have yet to be elucidated [47].

The production of this hormone by yeasts has often been reported using the Salkowski assay, which remains the most commonly used screening method. Specifically, strains such as *S*. *terrea*, *Rhodotorula babjevae*, *Williopsis saturnus*, *Meyerozyma guilliermondii*, *Lachancea thermotolerans*, and *Hanseniaspora uvarum* can synthesize IAA in both the presence and absence of Trp, although production is significantly enhanced when this precursor is supplied in the medium [11, 48]. Metabolically, yeasts degrade Trp into nitrogen sources and indole‐3‐ethanol, which is subsequently excreted. Consequently, plant root exudates rich in Trp represent a vital carbon and nitrogen source, supporting yeast growth and maintenance. Despite these findings, the ecological role of IAA in these microorganisms, as well as the evolutionary rationale behind its biosynthesis, remains poorly understood. Some studies postulate that *Saccharomyces cerevisiae* utilizes IAA to antagonize competing microorganisms and secure rapid spatial colonization [49, 50].

#### 3.2.2. CKs

CKs play an important role in organ development, particularly in structures called meristems, which are characterized by the presence of stem cells capable of producing plant organs. In the case of the shoot meristem, which manages the production of leaves, stems and flowers, CKs promote cell division and inhibit cell differentiation. In contrast, in the root meristem, CKs are associated with promoting differentiation of meristematic cells and inhibiting cell division, contributing to the formation of the entire root system [51]. Regarding yeasts, there are two reports of them synthesizing CKs. Laten and Zahareas‐Doktor [52] investigated the origin and presence of a specific type of CK in two yeast species: *S*. *cerevisiae* and *Schizosaccharomyces pombe*. A comparison of CK quantities found between species, along with a nucleoside analysis, led to the conclusion that the studied yeasts possess their own biosynthetic pathway for producing these molecules [52]. A later study about the ability of natural strains to synthesize zeatin, a CK hormone, revealed that 55% of 76 studied wild strains produced zeatin, predominating *Sporobolomyces roseus* and *Taphrina* sp. Yeasts isolated from the Arctic–alpine zone showed a pronounced ability to produce zeatin, compared with those isolated from tropical or temperate climates [53]. It should be noted that both reports cited here confirm CK production by yeasts under laboratory conditions. It has not yet been demonstrated whether yeast‐derived CKs reach biologically active concentrations at the plant–microbe interface or if they directly modulate plant development following inoculation.

#### 3.2.3. ABA

A sesquiterpenoid involved in dormancy, seed germination, and the induction of desiccation tolerance. Perceived by receptors in embryo cells, the ABA signaling molecule is activated in response to abiotic stress, enhancing freezing tolerance and delaying radicle emergence [54]. Although much of the current research on yeast‐based biostimulants has focused on their ability to produce phytohormones such as IAA, recent advances have expanded this potential to include other key phytohormones relevant to agriculture.

No yeast strain has yet been reported to naturally produce this plant hormone. Nevertheless, recent efforts to engineer a *S*. *cerevisiae* strain for heterologous ABA production, by incorporating the *Botrytis cinerea* biosynthetic pathway, highlight the promise of using yeast as a sustainable and scalable platform for the agricultural production of stress‐mitigating phytohormones [55].

#### 3.2.4. GA

Participate in fruit development and ripening, as well as in seed germination and hypocotyl elongation under normal environmental conditions [42]. Although little is known about GA production among yeasts, one report identified *Metschnikowia pulcherrima* (ex‐former *Torula pulcherrima*) as a producer of GA‐like substance [56]. A separate study using extraction and spectrophotometric analysis showed that *S*. *cerevisiae* can produce GA_3_, reaching concentrations of approximately 800 *μ*g/mL [57]. In another research, *Y*. *lipolytica* was genetically engineered by coexpressing heterologous genes from *Arabidopsis thaliana* and *Fusarium fujikuroi* to produce GA [58]. Collectively, evidence for natural GA production by yeasts remains sparse and methodologically limited. The concentrations reported have not been validated through chromatographic methods, and no study has confirmed GA‐mediated growth effects in plants following yeast inoculation under controlled or field conditions.

### 3.3. Abiotic Stress Tolerance

#### 3.3.1. ACCD Activity

The existence of ACCD in the plant significantly reduces ethylene (ET) production and may contribute to the improvement of plant growth in response to various abiotic and biotic stresses [59].

Some yeasts could transform 1‐aminocyclopropane‐1‐carboxylate (ACC), a precursor of ET, into ammonia and *α*‐ketobutyrate via the enzyme ACCD [10, 48]. This activity interferes with the production of ET in plants, a key hormone for functions such as root growth, germination, and senescence [60]. However, in stressful situations, the production of ET increases, which can lead to negative effects such as early senescence and leaf abscission [48, 61]. Yeasts with ACCD activity have been reported to mitigate these effects under controlled conditions, with improvements in plant tolerance to salinity and drought documented primarily in pot and greenhouse experiments [62]. Direct confirmation under field conditions remains limited.

#### 3.3.2. Drought Stress

Alzandi and Naguib [63] reported that yeast application enhanced drought tolerance in corn seedlings, improving NPK content, osmolyte accumulation, and antioxidant enzyme activity in roots. These findings, however, were obtained in a pot experiment under controlled conditions using agricultural soil, and their extrapolation to open field drought scenarios has not been validated.

The most common way to use yeast‐derived products as biostimulants is through yeast extracts, given their high content of amino acids, vitamins, carbohydrates, and other metabolites. Hammad and Ali [64] evaluated foliar application of a *S*. *cerevisiae* cell‐free extract on wheat under drought stress and found increases in leaf water content, total soluble sugars, and NPK uptake compared with stressed untreated plants. This represents a biostimulant effect mediated by yeast‐derived compounds rather than by live microbial inoculation.

Beyond yeast extracts, some yeasts produce polyamines, compounds involved in osmotic adjustment, membrane stabilization, and protection against oxidative damage in plants. For example, *C*. *tropicalis* HY has been shown to produce polyamines in vitro, as confirmed by arginine decarboxylase activity assays [10], suggesting that polyamine‐producing yeasts may influence physiological processes relevant to drought tolerance. Additionally, yeasts capable of mobilizing phosphorus or producing siderophores may indirectly support plant performance under water deficit by improving nutrient acquisition and maintaining metabolic activity during stress. Taken together, yeasts and yeast‐derived products show potential to modulate plant responses to drought through improvements in osmotic balance, nutrient status, and antioxidant protection, though validation under field conditions remains a priority (see Section 7.1).

#### 3.3.3. Salt Stress

A recent study employed a consortium of three yeast species, *Naganishia uzbekistanensis*, *Papiliotrema terrestris*, and *Solicoccozyma phenolica*, as a strategy to alleviate salt stress in zucchini plants. The application of this yeast consortium under saline conditions resulted in a significant increase in plant dry biomass compared with untreated controls. Furthermore, the study highlighted the impact of yeast inoculation on the rhizosphere microbiota, noting that bacterial taxa, particularly *Bacillus*, exhibited the most significant variability and showed a negative correlation with yeast abundance [65].

For its part, Raklami et al. [37] conducted a screening of yeast strains isolated from an extremophilic environment in Morocco. Among the isolates, species belonging to the genera *Naganishia* and *Rhodotorula* showed tolerance to high salinity conditions, sustaining growth in media containing up to 2 M NaCl. The selected strains were subsequently evaluated for their plant growth–promoting potential under nonstress conditions, where positive effects on plant development were observed.

It should be noted that most reported biostimulant effects of yeasts have been demonstrated under controlled conditions. Evidence from replicated field trials remains scarce, and reported outcomes are frequently strain, crop, and environment‐dependent. Therefore, biostimulant mechanisms described here should be interpreted as context specific rather than universally applicable.

## 4. Biocontrol Mechanisms of Yeasts

Although evidence supporting the biocontrol activity of yeasts in postharvest systems and controlled environments is available, their performance under open‐field conditions remains less well characterized. Their antagonistic efficacy is highly context‐dependent, shaped by complex interactions with local microbial communities, fluctuating environmental conditions, and varying pathogen pressure.

Yeasts offer several advantages for biocontrol applications; however, although specific functional attributes have been documented, others remain poorly understood. A comprehensive understanding of the modes of action of antagonistic yeasts, together with their ecological interactions within the resident microbiome, is essential for evaluating biosafety, efficacy, and consistency across in vitro, in vivo, and field conditions. Ultimately, elucidating these dynamics is critical for the development of stable, reproducible, and scientifically validated commercial bioproducts [66, 67].

### 4.1. Competition for Nutrients and Space

A primary mechanism by which yeasts function as biostimulants and biopesticides is through competition for nutrients and space. This mode of action proves particularly effective in environments characterized by high microbial competition and nutrient scarcity [68].

#### 4.1.1. Biofilm Formation

Biofilms are highly organized microbial communities whose development is orchestrated by quorum sensing (QS). This intercellular signaling system, mediated by the accumulation of quorum‐sensing molecules (QSMs), triggers specific gene expression patterns associated with morphological plasticity and enhanced environmental stress tolerance [69]. On postharvest fruit surfaces, biofilm assembly is characterized by the secretion of an extracellular polymeric substance (EPS) matrix and increased adhesion. This complex architecture is proposed to establish a physical barrier that restricts phytopathogenic fungi from accessing host resources [66, 70].

The most compelling evidence for biofilm‐mediated biocontrol is currently restricted to postharvest settings. For instance, *C*. *tropicalis*, a recognized biocontrol agent against *Colletotrichum musae* in bananas, forms biofilms predominantly composed of yeast cells with sporadic hyphal filaments [71, 72]. Similarly, Oztekin and Karbancioglu‐Guler [73] attributed the antagonistic activity of *Metschnikowia fructicola* against *Penicillium digitatum* and *Penicillium expansum* in lemons to its prolific biofilm production. Interestingly, the relationship between biofilm quantity and antagonistic efficacy is not strictly linear. While *M*. *pulcherrima* exhibits robust biofilm production and *Candida lusitaniae* shows minimal formation, both species provide substantial protection against *P*. *expansum* [73, 74]. This discrepancy underscores that while in vitro biofilm formation is frequently used as a preliminary screening trait, it does not universally predict biocontrol success, highlighting the need for context‐specific functional validation.

#### 4.1.2. Siderophore Production

Iron is a limiting element in microbial competition, and its assimilation by yeasts depends on cellular physiology and the physicochemical properties of the surrounding environment, particularly pH [75]. Siderophores are low‐molecular‐weight organic compounds with high affinity for ferric iron. These chelating agents enable yeasts to sequester environmental iron, competitively excluding phytopathogens that lack the specific transporters required to internalize these foreign siderophore–iron complexes.

A well‐characterized mechanism involves species such as *Metschnikowia citriensis* and *M*. *pulcherrima*, which secrete pulcherriminic acid. This precursor acts as a high‐affinity chelator of ambient ferric iron, resulting in the formation of pulcherrimin, a water‐insoluble red pigment complex [Fe(III)‐pulcherriminic acid]. Although this localized nutrient depletion restricts the vegetative growth and biofilm assembly of competing fungal pathogens in vitro [76–78], its relative contribution under complex open‐field conditions remains difficult to quantify due to fluctuating iron bioavailability and potential cross‐feeding by the native microbiota [79, 80]. Nevertheless, in postharvest pathosystems, this iron‐competition strategy represents a relevant factor in the antagonistic efficacy of *M*. *pulcherrima* against *B*. *cinerea*, as demonstrated in both in vitro and in vivo apple wound assays [81]. Interestingly, studies utilizing mutants deficient in pulcherriminic acid production suggest that while iron depletion is a primary antagonistic driver, these mutants retain residual antifungal activity [15, 76].

### 4.2. Mycoparasitism

Yeasts can also act as predators of other fungi, and the ecological drivers for this behavior remain a subject of debate; it is hypothesized to function as a competitive exclusion strategy for limited spatial niches or as a compensatory response to nutrient deprivation. Although yeast mycoparasitism has been scarcely studied in yeasts, the interaction relies on direct physical contact and the targeted secretion of hydrolytic enzymes [66, 82].

Species within the *Saccharomycopsis* genus form appendages that adhere to and penetrate the hyphae and spores of *Penicillium* species, subsequently extracting intracellular nutrients [83]. A similar contact‐dependent mechanism was visualized in vitro for *W*. *anomalus*, which fused with the hyphal cell walls of olive anthracnose pathogens (*Colletotrichum* spp.), leading to cytoplasmic drainage and target cell death [84].

Moving beyond in vitro observations, translational evidence for mycoparasitism is currently strongest in postharvest pathosystems. *Y*. *lipolytica* exhibits adhesion to *P*. *digitatum* mycelia, suppressing its growth via the localized release of lytic enzymes, a mechanism validated in both in vitro and in vivo citrus assays [85]. A comparable pattern was observed in *Pseudozyma antarctica*, which directly inhibited *P*. *digitatum* and *P*. *italicum* while undergoing a dimorphic yeast‐to‐pseudohyphal transition during coincubation. Although this morphological shift is linked to altered adhesive properties and metabolite production [86], extrapolating these intricate, density‐dependent cellular interactions to broad agricultural environments requires caution. The physical stability of yeast‐pathogen attachments under field‐level mechanical stress and abiotic fluctuations remains unverified [87, 88].

### 4.3. Lytic Enzymes

As previously noted, the secretion of hydrolytic enzymes, primarily *β*‐1,3‐glucanases, chitinases, lipases, and proteases, is a mechanism associated with mycoparasitism. Given that *β*‐glucans, glycoproteins, and chitin constitute the fundamental structural scaffold of fungal cell walls, their integrity is vital against environmental stressors [89–91]. The enzymatic breakdown of these polymers by antagonistic yeasts triggers the progressive degradation of the phytopathogenic fungal cell wall, releasing assimilable carbon and nitrogen. This process could contribute to yeast survival while consequently inducing host cell death due to structural weakening or loss of cytoplasmic contents [92, 93].

Experimentally, the application of purified extracellular exo‐*β*‐1,3‐glucanase from *Candida oleophila* attenuated conidial germination and inhibited the proliferation of *P*. *expansum* in apples [94]. Similarly, Marsico et al. [95] identified robust extracellular pectinase and lipase activities in *L*. *thermotolerans* and *M*. *pulcherrima*, with the latter also secreting proteases. These enzymatic profiles correlated with a marked suppression of *B*. *cinerea* mycelial growth in both in vitro and in vivo grapefruit assays, even at low inoculum levels. Furthermore, *Candida stellimalicola* has been investigated as a potential biocontrol agent candidate for the preventive control of *P*. *italicum* in oranges; its antagonistic performance is attributed to the synergistic action of chitinase production and the yeast′s inherent killer phenotype [96].

### 4.4. Killer Toxins

Antagonism by yeasts is further mediated by the production of killer toxins (or zymocins), an extensively documented phenomenon first reported in the 1960s [97]. These extracellular proteins or glycoproteins selectively target sensitive microorganisms through a two‐step recognition process involving specific receptors on the target cell wall and plasma membrane [98]. Although the killer phenotype was initially associated with *S*. *cerevisiae*, recent research has identified this trait across a broad diversity of genera. Notably, these toxins exhibit broad‐spectrum activity, proving lethal not only to related yeast strains but also to various filamentous fungi. Toxin‐producing strains possess intrinsic immunity to their own secretome, which could contribute to their competitiveness in agroecological environments [99, 100].

The molecular basis of this phenotype was subsequently linked to a symbiotic association with cytoplasmic double‐stranded RNA (dsRNA) viruses. In the classical *Saccharomyces* model, this system typically comprises a helper virus (L‐A) and a satellite dsRNA (M). The M‐dsRNA encodes both the secreted toxins and the components that confer immunity, whereas the autonomous L‐A virus provides the capsid proteins and the RNA‐dependent RNA polymerase (RdRp) essential for viral maintenance. Beyond this viral framework, it is now recognized that killer toxin production can also be encoded by linear dsDNA plasmids or nuclear chromosomal genes, depending on the yeast species. Although the evolutionary origins of these systems remain unknown, the association is primarily maintained through vertical transmission during mitotic division, although horizontal transfer can occur during sexual conjugation [101–104].

Although the initial docking of these exotoxins follows a conserved pattern, first interacting with the cell wall and subsequently with the plasma membrane, their downstream mechanisms of action are remarkably heterogeneous. These lethal effects are exerted through diverse pathways, including membrane pore formation, cell wall degradation, ion channel modulation, inhibition of cell wall biosynthesis, induction of apoptosis, and cell cycle arrest [105–108]. Recent applications highlight the biocontrol potential of these toxins in various pathosystems. For instance, Tsekova et al. [109] demonstrated that *Zygosaccharomyces bailii* immobilized on electrospun poly(3‐hydroxybutyrate) (PHB) scaffolds generated prominent inhibition zones and induced a loss of fungal pigmentation in *Fusarium graminearum*. Similarly, *W*. *anomalus* strains possessing killer activity were identified as the most effective candidates for reducing brown rot incidence in peaches and plums [110]. Furthermore, Perez et al. [111] isolated indigenous killer yeasts and evaluated their efficacy against green and blue molds; their findings revealed that strains of *Pichia* and *Wickerhamomyces* significantly suppressed *P*. *digitatum* proliferation in wounded lemon tissues.

### 4.5. VOCs

Another alternative mechanism of antagonism that does not require direct contact between the biocontrol agent and the pathogen involves the emission of VOCs. VOCs are small molecules (< 300 Da) characterized by their low solubility in water, high vapor pressure, and low boiling point. VOCs production is specific to each species, and a single strain can produce multiple components. This production depends on factors such as nutrient availability, ambient temperature, growth phase, and presence of other microorganisms, among others. The purpose of these molecules is to act as chemical signals for cell communication, release carbon, and promote or inhibit microbial growth, depending on the context [112–114].

Among the molecules commonly reported as yeast‐derived products are mainly alcohols, organic acids, esters, aldehydes, benzene derivatives, hydrazines, and other compounds. The set of these volatile molecules is called the “volatilome” [115–117]. Although the mechanism of action of VOCs on pathogens is not fully understood, it has been observed that exposure to these molecules can cause damage to the cell wall and membrane, resulting in the loss of intracellular content and oxidative stress [117]. This was demonstrated in a study where *P*. *kudriavzevii* significantly reduced the growth of *Monascus purpureus* mycelium and spore germination. It was identified that the volatile compound 2‐phenylethanol, produced by the yeast, caused morphological damage to the fungal hyphae [118]. Li et al. [119] reported the identification of nine volatile components produced by *M*. *pulcherrima*, which demonstrated reduced infection by *B*. *cinerea* in blueberries and prolonged their shelf life during storage. Finally, in a study conducted on peaches, it was found that VOCs produced by *Candida pseudolambica*, such as 3‐methyl‐1‐butanol and 2‐phenylethanol, inhibited *B*. *cinerea* germination through morphological and ultrastructural changes [120].

### 4.6. Induction of Resistance

ISR in plants and systemic acquired resistance (SAR) are two different types of systemic resistance that can be conferred by microorganisms to host plants or postharvest products. When the plant is exposed to pathogens, chemical substances, or beneficial microorganisms, specific pattern‐recognition receptors such as pathogen‐associated molecular patterns (PAMPs) or damage‐associated molecular patterns (DAMPs) are activated. Some antagonistic yeasts can trigger the gene expression of these pathways by the production of some metabolites or resistance inducers, such as chitinases, glucanases, lyases, or related enzymes, to the plant′s immune response [121, 122].

Huang et al. [123] reported that *M*. *guilliermondii* was effective in the biocontrol of *Botryosphaeria dothidea* in apple assays. Their results showed that the application of the yeast induced the expression of resistance marker genes (PR1 and PR5) against pathogen invasion, increased the activity of antioxidant enzymes such as catalase (CAT), peroxidase (POD), and superoxide dismutase (SOD), mitigating oxidative stress. Additionally, an increase in enzymatic activity related to the plant′s immune response, such as phenylalanine ammonia‐lyase (PAL) and polyphenol oxidase (PPO), was observed, which contributed to promoting fruit resistance against *B*. *dothidea*. Similarly, Yaguchi et al. [124] evaluated the effect of cell wall extracts from *Saccharomyces pastorianus* in in vitro assays with rice cell cultures, as well as in vivo assays with *A*. *thaliana* seedlings. In cell cultures, application of the extracts induced the accumulation of plant resistance‐related compounds, such as azelaic acid (AzA) and phenylalanine (Phe), and reduced triacylglycerol (TG) levels, suggesting metabolic changes associated with the activation of defensive responses. Additionally, in the in vivo assay, an increase in similar compounds to those found in rice cells was observed, and an improvement in plant resistance to *B*. *cinerea* infection was noted [123, 124].

Despite the documented potential of yeasts as biocontrol agents, several constraints limit their broader implementation. Mechanistic understanding remains incomplete for many of the interactions described above, particularly under open field conditions where ecological complexity reduces the predictability of biocontrol outcomes. Regulatory pathways for microbial biopesticides vary substantially across regions and require robust efficacy and safety data that are not yet available for most candidate strains. The growing pressure to reduce synthetic pesticide use provides an important incentive for advancing this field, but progress will require standardized evaluation protocols, better mechanistic characterization, and field‐level validation across diverse pathosystems [15].

## 5. Proposed Molecular Pathways: Evidence From Yeast–Fruit and Yeast–Root Systems

Yeast–plant interactions have been associated with molecular reprogramming in the host, spanning hormone signaling, nutrient acquisition, and defense‐related gene expression. Table 1 summarizes the reported mechanisms alongside their corresponding evidence tier. As the table shows, evidence for growth‐related pathways (hormonal modulation and nutrient acquisition) includes a small number of direct root‐level studies, whereas evidence for defense‐related gene expression derives almost entirely from postharvest fruit systems rather than from soil‐grown plants under agronomic conditions (Figures 2 and 3). The framework presented in this section should therefore be read as a set of working hypotheses, with growth‐ and defense‐related pathways resting on markedly different evidentiary foundations.

**Table 1:** Representative yeast‐mediated biocontrol and biostimulant mechanisms, associated genes/pathways, and their corresponding evidence level.

Strategy	Mechanism	Associated genes/pathways	Molecule or pathway involved	Evidence level	Reference
Biocontrol	Induction of resistance	*PR1* and *PR5*	Increased activity of CAT, POD, SOD, PAL, and PPO	In vivo—postharvest (apple)	[123]
Biocontrol	Induction of resistance	*Rbo*, *MAPK*, *MAPKK*, *CHI*, *PAL*, *CHS*, and *4CL*	Genes involved in biotic/abiotic stress response, signaling, defense, and secondary metabolism	In vivo—postharvest (citrus)	[125]
Biocontrol	Induction of resistance	*PAD* and *POD*	ROS regulation and phenylpropanoid pathway	In vivo—postharvest (kiwifruit)	[126]
Biocontrol	Induction of resistance	Genes related to ROS (RBOHD), CaM and CML, WRKY33, genes related to glutathione metabolism	SA, JA, and ET pathways; genes related to plant–pathogen interactions	In vivo—postharvest (mandarin)	[127]
Biostimulant	Stimulation of nitrate transporter gene expression	*NRT1*.*2*, *NRT2*.*1*, and *NRT2*.*3*	VOCs produced during yeast–tomato root interaction enhance nitrogen use efficiency	In vivo—tomato root (substrate, nonfield)	[31]
Biostimulant	Abiotic stress resistance	*HRGPs*, *DELLAs*, and *ABC*	Cell wall glycoproteins involved in abiotic stress response, plant growth, and development; gibberellic acid pathway	In vivo—greenhouse, multilocation (soybean)	[16]
Biostimulant	Drought stress alleviation	13 *ZmLEA* genes	Downregulation associated with reduced oxidative stress (H_2_O_2_, MDA); not attributable to yeast‐derived IAA or proline	In vivo—growth chamber/pot (maize, horticultural substrate)	[128]

**Figure 2:**
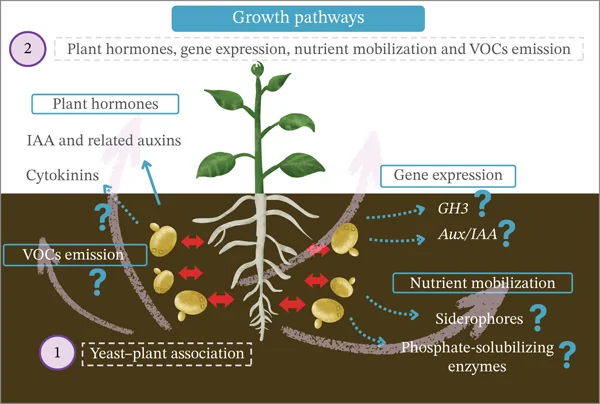
Yeast‐associated growth pathways in plants. The diagram summarizes the main growth‐related outputs associated with yeast–plant interactions in the rhizosphere. Purple numbers indicate the reading order: (1) yeast–plant association at the root interface and (2) downstream growth pathways involving plant hormone modulation, gene expression, nutrient mobilization, and VOC emission. Solid lines and bold labels indicate mechanisms directly supported in yeast–plant systems; dashed lines and question marks indicate mechanisms that remain putative or not yet directly demonstrated. Abbreviations: IAA, indole‐3‐acetic acid; VOCs, volatile organic compounds; GH3, Gretchen Hagen 3 auxin‐responsive genes; Aux/IAA, auxin/indole‐3‐acetic acid transcriptional repressors.

**Figure 3:**
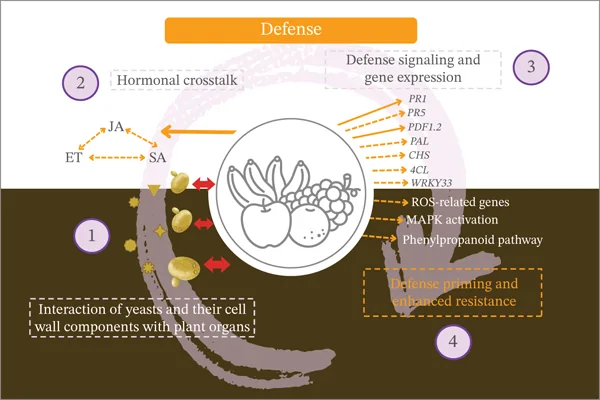
Yeast‐mediated defense responses reported in postharvest fruit systems. Schematic representation of the main defense‐related responses associated with yeast–fruit interactions: (1) interaction of yeasts and their cell wall components with fruit tissues; (2) hormonal crosstalk involving salicylic acid (SA), jasmonic acid (JA), and ethylene (ET); (3) defense signaling and modulation of defense‐related genes and pathways, including *PR1*, *PR5*, *PDF1*.*2*, *PAL*, *CHS*, *4CL*, and *WRKY33*, ROS‐related genes, MAPK activation, and the phenylpropanoid pathway; and (4) defense priming and enhanced resistance. Solid arrows represent relationships supported by in vivo yeast–fruit evidence, whereas dashed arrows indicate proposed or indirectly supported relationships. The evidence summarized here derives primarily from postharvest fruit systems, including citrus, apple, kiwifruit, and mandarin, and should not be extrapolated directly to soil‐grown plants without experimental validation. Abbreviations: SA, salicylic acid; JA, jasmonic acid; ET, ethylene; PR, pathogenesis‐related; PDF1.2, plant defensin 1.2; PAL, phenylalanine ammonia‐lyase; CHS, chalcone synthase; 4CL, 4‐coumarate‐CoA ligase; WRKY33, WRKY transcription factor 33; ROS, reactive oxygen species; MAPK, mitogen‐activated protein kinase.

Figure 2 translates the growth‐related mechanisms listed in Table 1 (hormonal modulation and nutrient acquisition) into a visual pathway, distinguishing outputs with direct in vivo support from those that remain putative.

### 5.1. Hormonal Modulation

Auxin‐ and CK‐producing yeasts have been associated with upregulation of hormone‐responsive genes (e.g., Aux/IAA and GH3) and with effects on root architecture and nutrient uptake under controlled inoculation [53, 129]. However, whether yeast‐derived hormones reach concentrations sufficient to trigger these responses in planta has not been directly confirmed through in planta hormone quantification for most reported cases.

### 5.2. Nutrient Acquisition

Siderophore secretion and phosphate solubilization have been linked to improved iron and phosphorus availability, with reported downstream effects on chlorophyll synthesis and photosynthetic performance [129]. VOCs produced by *S*. *terrea* modulate nitrate‐transporter genes (*NRT1.2*, *NRT2.1*, and *NRT2.3*) and stimulate lateral root formation in tomato, one of the few cases examined directly at the root level rather than inferred from other systems [31].

#### 5.2.1. Abiotic‐Stress–Related Gene Expression

A separate line of evidence links yeast inoculation to the downregulation of stress‐responsive genes rather than their induction. In maize seedlings grown under drought, inoculation with *Papiliotrema laurentii* downregulated 13 ZmLEA genes relative to untreated drought‐stressed plants, alongside reduced H_2_O_2_ and malondialdehyde concentrations in roots. Notably, the authors found this effect was not attributable to yeast‐derived IAA or to changes in proline accumulation, illustrating that a plausible hormonal mechanism can be directly tested and ruled out rather than assumed [128]. This is one of the few studies in this section conducted on whole, soil‐grown plants rather than isolated tissue or postharvest fruit, though it remains a growth‐chamber/pot system rather than a field trial.

Figure 3 visualizes the defense‐related mechanisms summarized in Table 1, highlighting their postharvest origin.

### 5.3. Defense‐Related Gene Expression

Yeast‐induced resistance does not follow a strict ISR/SAR division; available evidence points to overlapping, context‐dependent activation of SA, JA, and ET signaling. As Figure 3 highlights, direct support comes almost entirely from postharvest fruit systems: MAPK activation and PAL/CHS/4CL induction in citrus treated with *M*. *fructicola* [125], PR1/PR5 induction alongside antioxidant enzyme activity in apple treated with *M*. *guilliermondii* [123], ROS regulation and phenylpropanoid pathway activation in kiwifruit [126], and WRKY33/ROS‐related gene modulation in mandarin [127]. These findings establish that yeast treatment can activate defense signaling in harvested fruit tissue, but they do not demonstrate the same response in living, soil‐grown plants.

Evidence in whole‐plant systems is comparatively scarce and mostly derives from nonliving elicitors rather than live inoculation: yeast cell‐wall extracts (MAMPs) upregulate PDF1.2 and PR‐1 in *A*. *thaliana* and *Brassica* crops, and JA‐pathway metabolites in rice cell cultures [124, 130]. The clearest exception, and the only field‐level demonstration in this section, is *Pseudozyma churashimaensis*, a live leaf‐colonizing yeast that induced SA/JA/ET marker genes in pepper under both greenhouse and open‐field conditions [131].

Taken together, the molecular framework proposed here is a working model grounded in a small set of yeast‐specific studies. Growth‐related pathways rest on a limited but direct evidence base in roots and whole plants, whereas defense‐related pathways rest almost entirely on postharvest fruit systems; both should guide future hypothesis‐driven research on soil‐grown plants rather than be read as a consolidated account of yeast–plant molecular interaction. Section 6 examines how this evidence base translates into commercial applications.

## 6. Implications and Applications of Yeasts in Agroindustry

The potential advantages of biofertilizers and biopesticides include their renewable nutrient sources, maintenance of soil health and microbiota, reduction of synthetic fertilizer use by 25%–30%, environmental compatibility, absence of pest resistance, and cost‐effectiveness [17, 132]. However, several key technical aspects must be addressed to evaluate their commercial potential. Yeasts must demonstrate high and consistent efficacy in their intended ecological role, genetic stability, resistance to chemical or physical treatments, absence of harmful secondary metabolites, and stability under storage conditions. Another crucial factor is the formulation of yeasts themselves. As with other microorganisms, optimal formulations should promote rapid growth, appropriate particle size, and tolerance to light and temperature [17, 132]. Table 2 presents some examples of biostimulant or biocontrol yeast species already incorporated into commercial products.

**Table 2:** List of some yeast species with potential use in the agricultural industry.

Species	Commercial name	Function	Type of crop	Regulatory status	Reference
*C*. *oleophila* strain O	Nexy	Postharvest biocontrol	Apples, pears	Registered (US EPA)	[133]
*S*. *cerevisiae* LAS117	Romeo	Systemic resistance inducer (cell‐wall derivative, nonliving)	Grapevines, vegetables	Registered (European Union)	[134]
*S*. *cerevisiae* LAS02	Julietta	Biocontrol (live cells)	Grapevines, vegetables	Registered (European Union, low‐risk)	[135, 136]
*A*. *pullulans* DSM 14940/14941	Blossom Protect, Botector, BoniProtect	Field and storage disease control	Apples, pears, grapevines, strawberries	Registered (European Union, Canada)	[137, 138]
*M*. *fructicola* NRRL Y‐27328	Noli, Shemer	Biocontrol of *B*. *cinerea*, *Monilinia* spp.	Stone fruit, grapes, strawberries	Registered (European Union); potential sensitizer flagged	[139, 140]
*C*. *tropicalis* HY	BioGro 2	Phosphate solubilization; plant growth promotion	Rice	Registered (Vietnam); field‐validated	[10]
*B*. *subtilis* UYBC38 + *M*. *pulcherrima* M26	Zimevit	Biocontrol of *B*. *cinerea*	Grapevines	Field‐evaluated; registration status unconfirmed	[141]
*P*. *terrestris* PT22AV	YSY	Broad‐spectrum pathogen control	Multiple crops (unspecified)	Under regulatory review (European Union, United States); not yet registered	[142]
*C*. *oleophila* I‐182	Aspire	Postharvest biocontrol	Apples, pears, citrus	Discontinued	[143, 144]
*Cryptococcus albidus*	YieldPlus	Postharvest mold control	Strawberries, citrus, stone fruits	Discontinued	[143]
*Candida sake* CPA‐1	Candifruit	Postharvest biocontrol	Pome fruit	Discontinued	[143, 145]
*S*. *cerevisiae*	NutriSmart	Biofertilizer (N fixation, P solubilization, K mobilization)	Multiple crops	Company‐sourced information only; not independently verified	[146]

*Note:* The table was constructed based on commercial products whose main components are yeasts from different species, supported by scientific evidence on their mechanisms of biostimulation or biocontrol. Products are ordered by regulatory standing. Products for which information derives exclusively from company sources are flagged accordingly. Discontinued products are identified based on reasons reported in the literature. Products under regulatory review are noted as such.

The commercial products listed in Table 2 differ substantially in the strength of their scientific support and regulatory standing. They are discussed here in two groups: products with peer‐reviewed validation and formal regulatory registration, and products with discontinued status, limited documentation, or information derived exclusively from commercial sources. It should be noted that even within the first group, the nature and depth of supporting evidence vary, ranging from field‐validated efficacy to regulatory registration supported primarily by dossier‐based assessments rather than independent peer‐reviewed trials.

Among products with formal regulatory registration and published scientific support, BioGro (*C*. *tropicalis* HY) is the best supported example in field conditions, documented as a multistrain inoculant biofertilizer in Vietnamese rice systems where reductions in chemical fertilizer inputs were achieved without compromising yield in replicated field trials [10, 147]. Nexy contains *C*. *oleophila* strain O and holds US EPA regulatory approval as a postharvest biofungicide for apples and pears, with competition for nutrients and precolonization of wound sites described as its primary mode of action [133, 148]. Romeo contains cerevisane, a nonliving cell‐wall derivative of *S*. *cerevisiae* strain LAS117 registered in the European Union as a systemic resistance inducer, whereas Julietta contains live *S*. *cerevisiae* strain LAS02, approved in the European Union as a low‐risk active substance [134–136]. Blossom Protect, Botector, and BoniProtect are based on *A*. *pullulans* strains DSM 14940 and DSM 14941, registered in both the European Union and Canada; the Canadian PMRA‐approved Blossom Protect label specifies a formulation of 5 × 10^9^ CFU/g, and Botector holds full registration for suppression of Botrytis gray mold in grapes [137, 138]. Noli and Shemer are based on *M*. *fructicola* NRRL Y‐27328, approved as a fungicide in the European Union, although regulatory assessments flag the strain as a potential sensitizer requiring operator and worker protection measures [139, 140].

A second group of products requires more cautious interpretation. Aspire (*C*. *oleophila* I‐182), YieldPlus (*Cryptococcus albidus*), and Candifruit (*C*. *sake* CPA‐1) represent first‐generation postharvest biocontrol products that were commercialized and later discontinued due to business and marketing shortcomings [68, 143]. *C*. *sake* CPA‐1 retains support from formulation and semicommercial efficacy studies against postharvest pathogens of pome fruit [145, 149], but is no longer commercially available. YSY (*P*. *terrestris* PT22AV) is a patented yeast biocontrol agent currently undergoing regulatory review in the European Union and the United States and is not yet registered for commercial use [142]. Zimevit, a biofungicide combining *Bacillus subtilis* UYBC38 and *M*. *pulcherrima* M26, has been evaluated in field trials against grapevine gray mold caused by *B*. *cinerea* [141], but its current registration status remains unconfirmed in the published literature. Finally, for NutriSmart, strain‐level yeast identity could not be verified from peer‐reviewed or regulatory sources; available information derives exclusively from company product sheets and should not be treated as independently validated evidence [146].

Taken together, the products listed in Table 2 are supported by different types and depths of evidence. Nexy has direct product‐level support from nine semicommercial postharvest trials, seven of which reported disease‐incidence reductions ranging from 62 to 98%; however, these experiments provide semicommercial postharvest evidence rather than independent multilocation field validation [150]. Formulations based on *A*. *pullulans* have also been evaluated under commercial production conditions, although the results differ among products and pathosystems. Blossom Protect was assessed in replicated orchard trials for fire blight control, whereas Botector was evaluated in 26 on‐farm trials conducted over 3 years and nine locations; for Botector specifically, the estimated control of grapevine gray mold was significant but uncertain and varied among site‐years [151, 152]. BioGro is supported at the field level as a complete multistrain consortium, and its reported agronomic effects therefore cannot be attributed specifically to *C*. *tropicalis* HY. By comparison, the evidence summarized here for Romeo and Julietta relies primarily on active‐substance characterization and regulatory assessment, supplemented in the case of cerevisane by molecular studies, rather than on comparable independent multisite product trials [135, 153, 154]. *M*. *fructicola* provides a further example of the distinction between regulatory approval and reproducible efficacy, because the EFSA assessment reported low and variable efficacy for the representative uses evaluated [155]. Historical or discontinued products, precommercial candidates, mixed formulations without component‐separation experiments, and entries documented primarily through manufacturer information should therefore not be interpreted as equivalent to currently marketed products supported by independent efficacy trials.

Research on yeast‐specific formulation methods remains limited, as most studies focus on microbial formulations more broadly. In the review by He et al. [66], several bioprotection strategies involving biocontrol yeasts are discussed, including the combination of yeasts with antimicrobial compounds to enhance their stability and efficacy. Additionally, preadaptation to abiotic stress conditions has been explored to improve the stress tolerance of yeasts. For instance, adaptive laboratory evolution (ALE), through the optimization of culture media with protective additives, has been shown to increase yeast viability and biocontrol performance under stress conditions [123]. In *C*. *oleophila* I‐182, for example, preconditioning with sugars such as maltose and lactose prevented ATP depletion under thermal stress, thereby preserving mitochondrial function and improving antagonistic activity against postharvest pathogens [156]. Similarly, a native strain of *Debaryomyces hansenii* pretreated with sublethal concentrations of hydrogen peroxide demonstrated faster adaptation to the host microenvironment and significantly enhanced biocontrol efficacy [157]. These findings underscore the potential of ALE‐based approaches to improve stress resilience in biocontrol yeasts, particularly through the modulation of oxidative stress responses involving key antioxidant enzymes such as SOD, CAT, and POD. Such strategies hold promise for enhancing the robustness and practical applicability of yeast‐based biocontrol agents in agricultural systems.

Although specific economic comparisons of yeast‐based preparations versus conventional chemical protection in preharvest applications remain limited, available studies suggest potential agronomic and economic benefits. For instance, the coapplication of cyanobacteria and yeast inoculants in wheat cultivation resulted in notable socioeconomic benefits by enabling a reduction of approximately 25%–50% in the required mineral nitrogen fertilizers, without compromising crop productivity [158]. Similarly, in rice, a 25% reduction in chemical fertilizers combined with foliar application of a yeast consortium led to significant improvements in plant growth, biochemical parameters, and yield. This study also contributes to expanding the application of phyllosphere yeasts as biofertilizers in sustainable agriculture [158, 159]. These examples support the growing interest in the study of yeasts with potential applications as biofertilizers or biopesticides, as highlighted by Hernández‐Fernández et al. [17].

Beyond efficacy, the environmental release of live yeast‐based products raises biosafety considerations that are insufficiently addressed in the current literature. Introduced yeast strains may interact with resident soil and phyllosphere microbiomes in ways that alter community composition, compete with native beneficial microorganisms, or persist beyond the intended application window. Although most genera proposed as bioinputs, including *Metschnikowia*, *Wickerhamomyces*, *Candida* and *Aureobasidium*, are generally considered low risk for humans and animals, their ecological impact in agricultural systems has rarely been systematically assessed. Regulatory frameworks for microbial bioinputs vary substantially across regions. The European Union evaluates active microbial substances under Regulation (EC) No. 1107/2009, whereas the United States Environmental Protection Agency assesses biopesticide registrations under FIFRA. Regulatory assessments have applied standard precautionary sensitization warnings to some strains; for instance, EFSA [155] noted that *M*. *fructicola* NRRL Y‐27328 may have the potential to provoke sensitizing reactions, a precautionary classification routinely applied to microbial active substances in the absence of specific sensitization data. Future development of yeast‐based bioinputs should include ecological risk assessment, monitoring of inoculant persistence and dispersal, and transparent reporting of regulatory status as standard components of the commercialization pathway.

## 7. Current Challenges and Knowledge Gaps in Yeast Research

### 7.1. Laboratory Versus Greenhouse Versus Field Evidence

The evidence supporting yeast‐mediated plant benefits varies depending on the experimental system in which it was generated, and this heterogeneity has important consequences for interpreting the mechanisms described throughout this review.

Most of the biostimulatory characteristics reported for yeasts, including phosphate and potassium solubilization, ammonia production, IAA and CK synthesis, ACCD activity, and siderophore secretion, have been demonstrated primarily as strain screenings under in vitro conditions, typically in liquid cultures or on selective solid media. Although these assays confirm the biochemical potential of a given strain, they provide limited information on whether the same activity occurs at ecologically relevant concentrations in soil or rhizosphere environments, where pH, organic matter content, competing microorganisms, and nutrient availability can profoundly alter yeast physiology and function. For example, Yan et al. [24] observed that *C*. *mycoderma* produced opposite effects on soluble phosphorus depending on whether it was inoculated into natural red soil or permanently fertilized paddy soil, highlighting how contrasting soil conditions can reverse in vitro results.

Greenhouse and pot experiments represent an intermediate level of evidence. They confirm that in vitro characteristics can be translated into measurable plant responses under controlled inoculation, standardized soil substrates, and the absence of confounding agricultural management variables. Studies in this category have documented improvements in stem and root biomass, nutrient content, and stress tolerance indices in wheat, maize, tomato, zucchini, and other crops following yeast inoculation. However, these experimental systems rarely replicate the complexity of field soils, which harbor diverse and competitive native microbial communities that can suppress, dilute, or outcompete introduced yeast populations. The persistence and colonization capacity of inoculated strains under these conditions have rarely been monitored beyond the experimental period. Moreover, this greenhouse system is typically highly advanced and generally operates under tightly controlled climatic variables, ranging from temperature and humidity to the use of pots, while usually relying on the natural variation of light. Field‐level evidence on yeast‐based biostimulants remains scarce. Among the mechanisms reviewed, nutrient solubilization, particularly phosphate mobilization, has the most consistent support from field studies, partly because its outcomes (plant nutrient content, yield) are easier to quantify at an agronomic scale. Field‐proven examples include the multistrain inoculant BioGro containing *C*. *tropicalis* HY in Vietnamese rice systems, where reductions in chemical fertilizer use were achieved without compromising yield [10]. Evidence on hormone‐mediated growth promotion and abiotic stress relief under field conditions is largely indirect. Most studies report improvements in biomass or yield without measuring whether the proposed mechanism operates in the plant.

Even when testing spans multiple greenhouse locations, transcriptomic analysis is essential to verify whether the underlying mechanism remains consistent despite varying climatic conditions, a principle illustrated by Chambard et al. [16], who tested a yeast‐based biostimulant formulated with live cells on soybean crops grown in greenhouses across different locations, analyzing gene expression to determine whether the same abiotic‐stress‐tolerance pathways were activated regardless of potentially confounding climatic factors.

In the field of biocontrol, the evidence is comparatively stronger for postharvest applications, where the system is relatively closed, environmental variability is lower, and commercial products have been validated under semicommercial and regulatory conditions. Conversely, preharvest biocontrol in open field conditions has received less attention, and available data suggest that its efficacy may be inconsistent across growing seasons, varieties, and pathogen pressure levels. Products such as Blossom Protect (*A*. *pullulans*) and Noli/Shemer (*M*. *fructicola*) represent examples for which field data are available at the registration level, but the generalizability of these results to diverse agroecological zones has not yet been established.

Overall, the evidence on biostimulants and yeast‐based biocontrol agents should be understood as predominantly exploratory. In vitro and greenhouse findings are valuable for identifying candidate strains and mechanisms but are insufficient to predict agronomic performance. To overcome this gap, replicated multiyear field trials under commercially relevant conditions are required, with standardized protocols for measuring outcomes in both plants and microorganisms. Until such evidence is available for a broader range of strains and cultures, claims regarding performance and efficacy associated with yeast‐based bioinputs should be interpreted with due caution.

### 7.2. Strain‐Specific Versus Generalizable Effects

In general, the observed biostimulant effects tend to be strain‐specific. For instance, broad screenings for specific activities, such as IAA production, frequently reveal that between 70% and 100% of strains exhibit this capacity. However, these strains do not produce IAA at equivalent levels, nor do they respond uniformly under identical conditions [11, 12, 48]. This variability arises because each strain carries a genomic signature shaped by its native environment, where multiple ecological factors dictate its overall performance. For example, in a study on *Drosera* plants evaluating strains isolated from both the phyllosphere and rhizosphere, the highest levels of IAA production were observed in leaf‐derived strains compared with root‐derived ones. However, no statistically significant correlation was reported [12].

Similarly, strains originating from extreme environments may establish stronger, multimechanistic associations with plants. Conversely, a strain may display beneficial effects in its native context but fail to perform effectively when introduced into commercial agricultural systems. This highlights the critical need for future meta‐analyses and systematic comparative studies to rigorously quantify these variations. Specifically, data‐driven approaches that cross‐reference isolation origins (e.g., specific plant compartments) with in planta efficacy are required to determine whether robust ecological correlations can be established.

This strain dependency is also evident in the context of biocontrol. Although antagonistic capabilities are frequently generalized at the species level, the literature reveals significant intraspecific variability. A prime example is *M*. *pulcherrima*, a species for which biocontrol efficacy reports are notoriously contradictory. This discrepancy can be partially attributed to taxonomic complexities. As Sipiczki [160] highlights, *M*. *pulcherrima* and related pulcherrimin‐producing yeasts exhibit “fuzzy species boundaries,” suggesting that isolates classified under the same nomenclature may represent distinct cryptic lineages with varying antagonistic potentials. Beyond these taxonomic challenges, the underlying mechanisms driving biocontrol are inherently multifaceted. Studies utilizing targeted genetic modifications, such as the disruption of the *Snf2* gene, which regulates pulcherriminic acid biosynthesis in *M*. *pulcherrima*, demonstrated that even when the production of this primary antagonistic metabolite is suppressed, mutant strains retain residual suppressive activity [76]. This functional redundancy underscores that successful biocontrol is rarely the result of a single isolated mechanism; rather, it depends on a synergistic integration of multiple metabolic pathways unique to each highly adapted strain.

### 7.3. Confirmed Mechanisms Versus Proposed Hypotheses

Although numerous mechanisms have been proposed within the biostimulant and biocontrol domains, only a subset has been definitively confirmed to drive specific physiological responses in plants. This disconnect is particularly evident regarding yeast‐mediated phytohormonal effects. For instance, although auxin‐producing yeast strains are frequently hypothesized to enhance plant growth, few studies have quantified in planta IAA content to determine the extent to which this physiological response may be related to yeast‐derived hormone production. Furthermore, within the biocontrol domain, in vitro antagonism is frequently reported and is often associated with the production of lytic enzymes, siderophores, or VOCs; however, demonstrating the contribution of these mechanisms to pathogen suppression under complex field conditions remains challenging. Many studies extrapolate laboratory plate assay findings to agricultural settings without directly quantifying the in situ expression or concentration of these antifungal traits. Compounding this issue is the historical bias of yeast biocontrol research towards postharvest applications. Because controlled postharvest environments on fruits present fewer ecological variables, direct antagonistic mechanisms, such as competition or lytic enzyme activity, are considerably easier to validate than in open‐field crop systems. With the notable exception of ISR, which intrinsically relies on the active physiological response of a living plant, the exact mechanisms operating in the complex and dynamic phyllosphere or rhizosphere remain largely unconfirmed. Similarly, the application of yeasts to mitigate abiotic stress consistently results in enhanced plant vigor, yet the underlying mechanisms remain diverse and largely hypothetical, requiring further investigation. For this reason, authors such as those proposed by du Jardin [1] and Yakhin et al. [4] define biostimulants primarily by their observable agronomic effects rather than their precise molecular mechanisms of action.

In contrast, evidence supporting nutrient solubilization and enhanced nutrient availability is substantially more robust. These parameters are easier to quantify in both plant tissues and soil matrices, supported by a higher volume of field trials. Nevertheless, current literature still lacks comprehensive studies addressing environmental variability and the baseline presence of native yeast across distinct regions.

Future research must elucidate how yeast inoculation alters the native rhizosphere microbiome, alongside evaluating the long‐term viability of introduced yeast populations in soils, substrates, or hydroponic systems. Rigorous studies are required to determine establishment success, optimal application frequencies, and the persistence of metabolic activity. Ultimately, a critical question remains unanswered: Do introduced yeasts successfully colonize the rhizosphere and, if so, which specific ecological niches do they occupy?

### 7.4. Demonstrated Efficacy Versus Deployment Barriers

Beyond strain‐level variability, three additional constraints limit the practical deployment of yeast‐based products and are rarely addressed together in the literature: dose dependency, formulation/scalability, and host specificity. Efficacy is frequently concentration‐dependent rather than binary, and this holds for both biocontrol and biostimulant applications. On the biocontrol side, *Y*. *lipolytica* MBC25 suppressed *B*. *cinerea* in strawberries most effectively at 10^8^ CFU/mL, whereas 10^5^ CFU/mL produced no significant difference from the untreated control [161]. On the biostimulant side, *Pa*. *laurentii* alleviated drought stress in maize only at 10^7^ cells/mL among four tested concentrations (10^6^–10^9^ cells/mL), with no growth‐promoting effect observed in well‐watered plants at any concentration, illustrating that yeast‐mediated biostimulation can follow a nonmonotonic dose–response relationship rather than a simple more‐is‐better pattern [128]. Scalability and formulation present a separate set of obstacles: moving a strain from laboratory or greenhouse screening to a stable, marketable product requires solving cell viability during storage, delivery format, and cost‐effective mass production, constraints that a recent review identified as primary barriers limiting the commercial accessibility of antagonistic yeasts despite consistent laboratory‐level efficacy [162]. Host specificity compounds these limitations, as the same strain can perform inconsistently across crop species, cultivars, or tissue types, meaning that efficacy demonstrated in one pathosystem cannot be assumed to transfer to another without direct testing.

Recent work continues to expand this evidence base. Hernández‐Fernández et al. [163] profiled 25 epiphytic PGPY strains from *Vitis vinifera* and validated growth‐promoting effects in greenhouse trials, whereas Sharma et al. [164] provide a complementary synthesis of yeast‐mediated plant growth promotion mechanisms. Similarly, Makhokh et al. [165] developed a yeast‐based reporter system for screening drought‐tolerance biostimulants, and Abd El‐Sattar and Abdelhameed [166] demonstrated salt‐stress amelioration through foliar yeast extract application in *Vicia faba*. These recent studies reinforce the trajectory identified throughout this review, while underscoring that field‐scale validation remains the primary translational barrier.

Figure 4 provides a conceptual visual summary of the translational pipeline and major bottlenecks in agricultural yeast applications, mapping the progression from in vitro screening to field trials.

**Figure 4:**
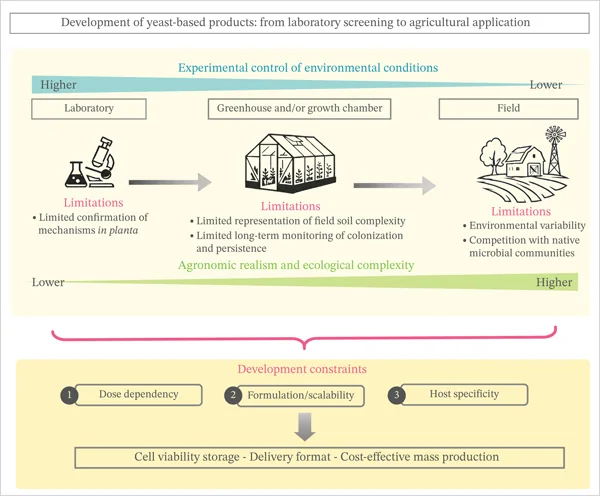
Conceptual framework for the development of yeast‐based agricultural products from laboratory screening to field application. The decreasing blue line illustrates the reduction in experimental control that occurs when moving from laboratory experiments to field trials. The increasing green line represents the rise in complexity and realism of agricultural conditions as experiments transition from laboratory to field. Each experimental stage presents specific limitations that can affect the interpretation and translation of yeast performance across scales. Key constraints throughout product development include dose dependency, formulation and scalability, and host specificity.

## 8. Conclusions

The evidence reviewed here supports a nuanced assessment of yeasts as agricultural microorganisms. They are promising in scope, uneven in experimental validation, and still far from the mechanistic consolidation required for reliable large‐scale application. This conclusion is not a dismissal of the field, but a precise characterization of where it stands.

Among the mechanisms reviewed, nutrient solubilization, iron competition, lytic enzyme secretion, and killer toxin production have the strongest experimental grounding, supported by direct in vitro evidence and, in several cases, by postharvest or greenhouse‐level confirmation. VOC‐mediated effects and defense gene induction occupy an intermediate position, as responses have been documented in planta but the causal agents, the specificity of the plant response, and the conditions under which effects are reproducible remain only partially established. By contrast, hormone‐mediated growth promotion and abiotic stress alleviation, among the most frequently reported yeast‐associated benefits, present a consistent gap between biochemical trait detection and plant‐level confirmation. In many studies, increased biomass or stress tolerance is reported without direct evidence that yeast‐derived hormones reached biologically active concentrations at the plant–microbe interface, or that the observed responses were causally linked to yeast activity rather than to indirect soil or microbiome effects.

A further tension runs through the literature, namely, the contrast between the taxonomic and functional diversity of the yeasts studied and the narrow range of experimental systems in which they have been evaluated. Most mechanistic evidence derives from postharvest fruit systems, *A*. *thaliana* models, or controlled pot experiments under optimal conditions. These systems have generated important foundational knowledge, but they are structurally different from the soil‐grown, field‐exposed crops in which yeast‐based products would ultimately need to function. The degree to which molecular responses documented in postharvest citrus or model plants translate to field‐grown cereals, legumes, or vegetables facing biotic stress, abiotic stress, or multifactorial environmental scenarios remains largely unknown. Resolving these tensions will require a deliberate shift in research design. Five priorities are proposed here. First, causal mechanism validation must replace trait screening as the dominant research mode, integrating mutant‐based approaches, in planta hormone quantification, and assays that distinguish the contributions of live cells, cell‐free extracts, VOCs, and purified metabolites. Second, strain‐level characterization, including genome‐based biosafety assessment and phenotypic stability evaluation, must become standard practice before strains are proposed for agricultural application. Third, the ecological behavior of inoculated yeasts in native microbiomes requires systematic investigation, particularly regarding persistence, colonization dynamics, and interactions with resident microbial communities. Fourth, field trials must be expanded using standardized designs across diverse agroecological conditions, including comparisons with conventional inputs and existing registered bioproducts. Fifth, formulation and commercialization research must address practical barriers including shelf life, carrier compatibility, registration requirements, and quality control that currently limit the translation of laboratory findings into deployable products.

The field is not yet at a stage where broad agricultural recommendations can be made. Yeasts are best understood at this point as a scientifically promising but experimentally incomplete resource. Their greatest near‐term contribution is likely to come from targeted applications, whether specific strains or cell‐wall derivatives in defined pathosystems or production contexts, rather than from broad‐spectrum deployment. A coordinated research agenda linking molecular biology, microbial ecology, field agronomy, and regulatory science is the necessary condition for moving yeast‐based bioinputs from experimental tools to reproducible, commercially viable technologies.

## Author Contributions

Conceptualization: M.C., C.S‐M., and L.G. Writing—original draft preparation: M.C., C.S‐M., and J.F. Writing—review and editing: L.G., M.C., C.S‐M., and J.F. Supervision: L.G.

## Funding

No funding was received for this manuscript.

## Disclosure

All authors have read and agreed to the published version of the manuscript.

## Consent

The authors have nothing to report.

## Conflicts of Interest

The authors declare no conflicts of interest.

## Data Availability

Data sharing is not applicable to this article as no datasets were generated or analyzed during the current study.
